# Supplementary material for: Saccharomyces boulardii (CNCM I-745) improves intestinal damage in sepsis by remodeling intestinal flora structure
Source: Front Cell Infect Microbiol. 2025 Jul 31;15:1602792. doi: 10.3389/fcimb.2025.1602792 (PMC12350329; doi:10.3389/fcimb.2025.1602792)
Supplement: Supplementary file 1 [file DataSheet1.zip › summary/summary/6_Taxonomy_community/5_Genus_phylotree/genus_phylotree_Group.pdf]

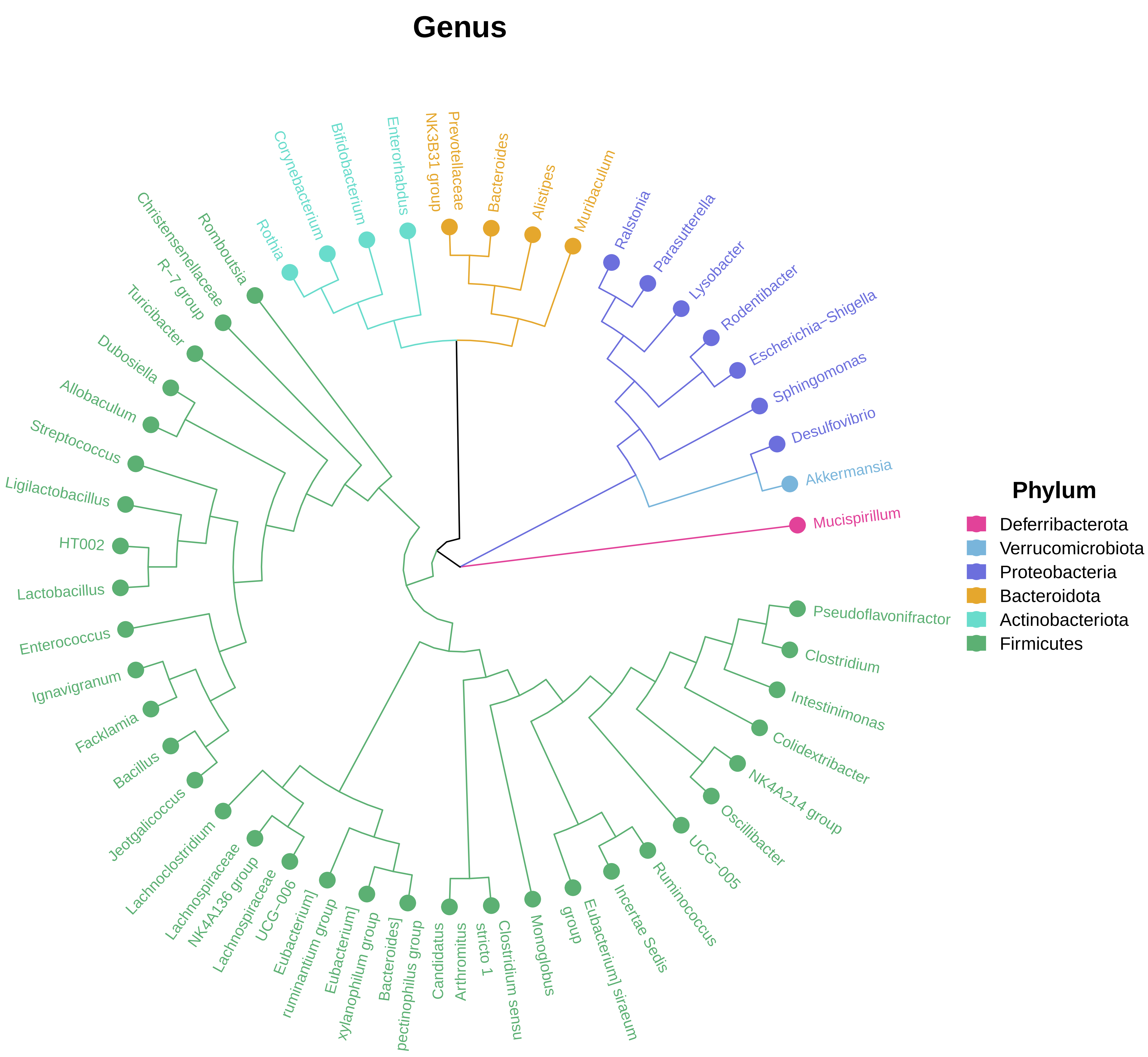
Genus
Rothia
Corynebacterium
Bifidobacterium
Enterorhabdus
NK3B31 group
Prevotellaceae
Bacteroides
Alistipes
Muribaculum
Ralstonia
Parasutterella
Lysobacter
Rodentibacter
Escherichia−Shigella
Sphingomonas
Desulfovibrio
Akkermansia
Mucispirillum
Pseudoflavonifractor
Clostridium
Intestinimonas
Colidextribacter
NK4A214 group
Oscillibacter
UCG−005
Ruminococcus
Incertae Sedis
Eubacterium] siraeum
group
Monoglobus
Clostridium sensu
stricto 1
Arthromitus
Candidatus
pectinophilus group
Bacteroides]
xylanophilum group
Eubacterium]
ruminantium group
Eubacterium]
UCG−006
Lachnospiraceae
NK4A136 group
Lachnospiraceae
Lachnoclostridium
Jeotgalicoccus
Bacillus
Facklamia
Ignavigranum
Enterococcus
Lactobacillus
HT002
Ligilactobacillus
Streptococcus
Allobaculum
Dubosiella
Turicibacter
R−7 group
Christensenellaceae
Romboutsia
Phylum
Deferribacterota
Verrucomicrobiota
Proteobacteria
Bacteroidota
Actinobacteriota
Firmicutes
